# *Origanum majorana* Essential Oil Triggers p38 MAPK-Mediated Protective Autophagy, Apoptosis, and Caspase-Dependent Cleavage of P70S6K in Colorectal Cancer Cells

**DOI:** 10.3390/biom10030412

**Published:** 2020-03-06

**Authors:** Khawlah Athamneh, Aysha Alneyadi, Halima Alsamri, Asma Alrashedi, Abdulrasheed Palakott, Khaled A. El-Tarabily, Ali H. Eid, Yusra Al Dhaheri, Rabah Iratni

**Affiliations:** 1 Department of Biology, College of Science, UAE University, United Arab Emirates University, Al-Ain P.O. Box 15551, UAE; 200834888@uaeu.ac.ae (K.A.); 200907889@uaeu.ac.ae (A.A.); 200813902@uaeu.ac.ae (H.A.); 201003061@uaeu.ac.ae (A.A.); abdulrasheed1984@uaeu.ac.ae (A.P.); ktarabily@uaeu.ac.ae (K.A.E.-T.); yusra.aldhaheri@uaeu.ac.ae (Y.A.D.); 2 Khalifa Center for Genetic Engineering and Biotechnology, United Arab Emirates University, Al-Ain P.O. Box 15551, UAE; 3 Department of Pharmacology and Toxicology, Faculty of Medicine, American University of Beirut, Beirut 1107 2020, Lebanon; ae81@aub.edu.lb

**Keywords:** *Origanum majorana*, colon cancer, autophagy, apoptosis, p38MAPK, p70S6K

## Abstract

Colorectal cancer (CRC) is the third most common type of cancer in terms of incidence and mortality worldwide. Here we have investigated the anti-colon cancer potential of *Origanum majorana* essential oil (OMEO) and its underlying mechanisms of action. We showed that OMEO significantly inhibited the cellular viability and colony growth of human HT-29 colorectal cancer cells. OMEO induced protective autophagy, associated with downregulation of the mTOR/p70S6K pathway, and activated caspase-8 and caspase-9-dependent apoptosis. Blockade of autophagy with 3-methyladenine (3-MA) and chloroquine (CQ), two autophagy inhibitors, potentiated the OMEO-induced apoptotic cell death. Inversely, inhibition of apoptosis with the pan-caspase inhibitor, Z-VAD-FMK, significantly reduced cell death, suggesting that apoptosis represents the main mechanism of OMEO-induced cell death. Mechanistically, we found that OMEO induces protective autophagy and apoptotic cells death via the activation of the p38 MAPK signaling pathway. Pharmacological inhibition of p38 MAPK by the p38 inhibitors SB 202190 and SB 203580 not only significantly decreased apoptotic cell death, but also reduced the autophagy level in OMEO treated HT-29 cells. Strikingly, we found that OMEO also induces p38 MAPK-mediated caspase-dependent cleavage of p70S6K, a protein reported to be overexpressed in colon cancer and associated with drug resistance. Our findings suggest that OMEO inhibits colon cancer through p38 MAPK-mediated protective autophagy and apoptosis associated with caspase-dependent cleavage of p70S6K. To the best of our knowledge, this study is the first to report on the implications of the p38 MAPK signaling pathway in targeting p70S6K to caspase cleavage.

## 1. Introduction

Colorectal cancer (CRC) is the third most common type of cancer in terms of incidence and mortality, and affects both sexes almost equally [1]. CRC used to be rather uncommon back in the 1950s. However, it has seen an increase in incidence in the past six decades and has now become one of the predominant cancers (the third most common type); it accounts for approximately 10% of all cancer-related mortalities. Reasons that might explain the alarmingly increased incidence include an aging population (most patients diagnosed with CRC are age 50 and older), poor diet and lifestyle, smoking, a low rate of physical activity, and obesity [1]. CRC is a complex disease; it usually grows in the lining of the colon and the rectum in the form of a polyp, which is a mass bulging in the lumen. Not all the polyps are neoplastic, nor do all of them develop into cancer. However, the majority of CRC evolves from adenomatous polyps [2].

For thousands of years, humankind has looked to plants for their medicinal benefits. Plants were used, starting from the leaves and moving to the roots, and extracted as crude extracts such as tinctures, teas, powders, and other forms of formulations [3,4]. Most agents in chemotherapy today are linked to natural compounds. About three-quarters of the chemotherapeutic agents introduced since 1940 are in fact natural compounds or are direct derivatives. Irinotecan, etoposide, and paclitaxel are examples of plant-derived compounds used in cancer treatment, and many other compounds are in clinical trials [5,6,7].

Plant-derived essential oils (EO) are complex mixtures made of low-molecular-weight compounds mostly extracted by steam distillation; two of their primary ingredients, terpenoids, and phenylpropanoids, provide essential oils’ characteristic aromatic and biological properties. Essential oils carry out a variety of pharmaceutical and biological activities, including antibacterial, antifungal, anticancer, antimutagenic, antidiabetic, antiviral, anti-inflammatory, and antiprotozoal [8,9,10]. In addition, essential oils have been shown to possess antioxidant and insect-repellent activities [9,11].

*Origanum majorana* is an herbaceous plant found in southern Europe and the Mediterranean area. The herb, from the family Lamiaceae and commonly known as marjoram, can grow up to 60 cm. *O. majorana* is widely used as a garnish in food preparation, as well as being a medicinal plant used for different purposes in the traditional medicine of different regions. Studies on this plant resulted in the identification of many of its active compounds. Notably, marjoram is rich in polyphenols such as flavonoids, which are bioactive compounds that, potentially, have beneficial pharmacological activities; in addition, it contains phenolic terpenoids, oxygenated monoterpene, tannins, and phenolic glycosides [12]. Some of the pharmacological activities that *O. majorana* leaves have been recorded to possess are antioxidant [13], antimicrobial [14,15,16], antineurodegenerative [14], and anticancer properties [17,18,19]. In addition, *O. majorana* extract was also reported to inhibit platelet adhesion, aggregation, and secretion [20]; it attenuated the nephrotoxicity of cisplatin anticancer drug [21], reduced the occurrence of ulcers, and replenished the depleted gastric wall mucus [22]. Our group has previously showed that *O. majorana* ethanolic extract (OMEE) has a significant effect on triple-negative breast cancer cells, promotes mitotic arrest at G2/M phase, induces apoptosis, and inhibits migration and metastasis [17,18]. In addition, we showed that ethanolic extract inhibited colon cancer cells in vitro and in vivo through the induction of abortive autophagy, with subsequent activation of apoptosis [19].

In addition to its antimicrobial and antifungal properties, *O. majorana* essential oil (OMEO) was reported to be nontoxic. Indeed, Wistar rats treated for 14 days with a dose of 2 g/kg body weight showed no sign of toxicity and no difference in body weight between OMEO-treated and control rats [23]. OMEO was also shown to attenuate toxic effects such as oxidative damage and liver injury of prallethrin in rats [24]. Studies also reported that OMEO was nonirritant, nonsensitizing, and nonmutagenic [25]. Despite all these reports, and unlike the alcoholic extract, data regarding the potential anticancer activity of OMEO are still lacking. Here we decided to examine the activity of OMEO against human colon cancer cells. Our results demonstrate that OMEO inhibits the growth of colon cancer cells in vitro and in vivo through p38 MAPK-induced apoptosis.

## 2. Material and Methods

### 2.1. Origanum majorana Essential Oil

The *Origanum majorana* essential oil (Figure S1) used in this study was obtained from PRANARÔM Scientific Aromatherapy, commercially available in pharmacies (Montpellier, France).

### 2.2. Cell Culture, Chemicals, and Antibodies

Human colon cancer cells HT-29 (Cat# 300215) were purchased from CLS (Cell Lines Service, Eppelheim, Germany) and were maintained in DMEM supplemented with 10% heat inactivated fetal bovine serum (FBS), 100 U/mL penicillin/streptomycin (Hyclone, Cramlington, UK). Antibodies against caspase-8 and p27 were obtained from Cell Signaling (Cell Signaling Technology, Danvers, MA, USA); those raised against β-actin were obtained from Santa Cruz Biotechnology (Santa Cruz Biotechnology, Santa Cruz, CA, USA). Antibodies raised against Cleaved PARP were obtained from Abcam (Abcam, Cambridge, UK); those raised against p21 and γH2AX were obtained from Millipore (Millipore, Hayward, CA, USA). Chloroquine (CQ) was obtained from Sigma-Aldrich (Sigma-Aldrich, Saint-Quentin Fallavier, France); 3-Methyladenine and Z-VAD-FMK were obtained from Millipore, SB 203,580 was purchased from Cell Signaling and SB 202190 was purchased from Abcam. Antibodies against caspase 8, cleaved caspase 3, mTOR, phospho-mTOR, p70S6, phosphor-p70S6, p38, phosphor-p38, LC3, and Beclin-1 were obtained from Cell Signaling; those against γH2AX were obtained from Millipore, those against TNFα, p62/SQSTMI, and cleaved PARP were obtained from Abcam. The antibody against β-actin was obtained from Santa Cruz Biotechnology.

### 2.3. Measurement of Cellular Viability

HT-29 cells were seeded in 96-well plates in triplicate at a density of 7000 cells per well. Twenty-four hours after the seeding, cells were treated with or without varying dilutions of OMEO for 6, 24, and 48 h. The dilutions used and their corresponding concentrations are as follows: 0.01% (64 μg/mL), 0.02% (128 μg/mL), 0.04% (256 μg/mL), and 0.1% (640 μg/mL). Following treatment, cellular viability of HT-29 cells was assessed with a Colorimetric Cell Cytotoxicity Assay Kit, ab112118 (Abcam), according to the manufacturer’s instructions. The absorbance was measured at 570 nm and 605 nm, with the ratio OD_570nm_/OD_605nm_ being proportional to the number of viable cells. The results are illustrative of an average of four independent experiments. Data are presented as proportional viability (%) by comparing the OMEO-treated group with the untreated cells, the viability of which is assumed to be 100%.

### 2.4. HPLC-MS

The components of *Origanum majorana* essential oil was analyzed using LC-MS (6420 Triple Quadrupole, Agilent Technologies, Santa Clara, CA, USA). OMEO was filtered using a 0.45-um syringe filter preceding the analyses. The sample was analyzed by an Agilent EclipsePlus-C18 column (1.8 μm particle size, 2.1 mm × 50 mm, Agilent Technologies) maintained at 35 °C, coupled to a tunable UV-Vis detector and 6420 Triple Quadrupole LC/MS System (Agilent Technologies, Santa Clara, CA, USA). 0.1% formic acid and acetonitrile were used as mobile phase according to the following schedule: 0–2.5 min: 0% B, 2.5–15 min: 20%–100% B, 15–18min: 100% B and 18–25 min: 5% B with 0.2 mL/min. An electrospray ionization (ESI) source was used in LC-MS system in positive polarity. The LC-MS operating conditions were as follows: capillary voltage: 4 kV, nebulizer pressure: 45 psi, drying gas flow: 11 L/min, and drying temperature 325 °C. The mass range monitored was 100 to 1000 Da.

### 2.5. Colony Formation Assay

HT-29 cell-line cells were cultured in six-well plates at a density of 1000 cells per well and permitted to grow for about 13 days to form colonies before OMEO treatment was added to a freshly prepared growth medium, and the colonies were allowed to grow for three additional days. Next, colonies were washed three times with 1X phosphate buffer saline (PBS), then fixed for 15 min with 4% paraformaldehyde and stained with 0.01% crystal violet for 30 min. Colonies were counted and their surface area was determined by using ImageJ software (https://imagej.nih.gov/ij/download.html).

Colony formation assay was also assessed when HT-29 cells were allowed to form colonies in the presence of media with or without various concentrations of OMEO for 13 days and then stained as described above.

### 2.6. Whole Cell Extract and Western Blot Analysis

HT-29 cells (3 × 10^6^) were seeded in 100-mm culture dishes and cultured for 24 h prior to treatment. After incubation with OMEO for the specific durations, cells were washed twice with ice-cold PBS, scraped, pelleted, and lysed in a RIPA buffer (Thermo Scientific, Rockford, IL, USA) complemented with phosphatase inhibitor (Roche, Mannheim, Germany) and protease inhibitor cocktail (Roche, Mannheim, Germany). After that, cell lysate was incubated for 30 min on ice, then centrifuged at 14,000 rpm at 4 °C for 20 min. A BCA protein assay kit (Thermo Scientific, Rockford, IL, USA) was used to determine the protein concentration of lysates. Cell lysates were then aliquoted in 30 µg and resolved onto 6%–15% SDS-PAGE along with Page Ruler Plus Pre-stained Protein Ladder (Thermo Scientific). Proteins were transferred onto PVDF membranes (Thermo Scientific) and blocked for 1 h at room temperature with 5% non-fat dry milk prepared in TBST (TBS and 0.01% Tween 20). Later, PVDF membranes were incubated with specific primary antibodies in blocking buffer overnight at 4 °C. Horseradish peroxidase-conjugated anti-IgG was used as the secondary antibody. Immunoreactive bands were detected by ECL chemiluminescent substrate (Thermo Scientific), and FEMTO and chemiluminescence were detected using the LiCOR C-DiGit blot scanner. Where needed, membranes were stripped in Restore Western blot stripping buffer (Thermo Scientific) as per the manufacturer’s instructions. Western blots are representative of at least two independent experiments.

### 2.7. Statistical Analysis

Data were described as group mean ± SEM. The data were evaluated via Student’s *t*-test. Significance for all statistical comparisons was * *p* < 0.05, ** *p* < 0.005, *** *p* < 0.001.

## 3. Results

### 3.1. HPLC-MS Identification of Constituents in Origanum majorana Essential Oil

To confirm the identity of the commercial OMEO used in this study, the essential oil composition was analyzed by LC-MS and the identified compounds were compared with those reported in the literature. Representative LC-MS chromatograms and the compounds detected are shown in Figure S2A,B, respectively. As expected, compounds such as Terpinen-4-ol, alpha-Terpinol, Alpha-Pinene, Camphene, p-Cymol, B-Caryophyllene, Bicyclogermacrene and Neophytadiene were present in OMEO, in agreement with previous reports [15,26,27,28,29].

### 3.2. Origanum majorana Essential Oil Inhibits the Cellular Viability and Colony Growth of Human Colorectal Cancer Cells

**Figure 1:**
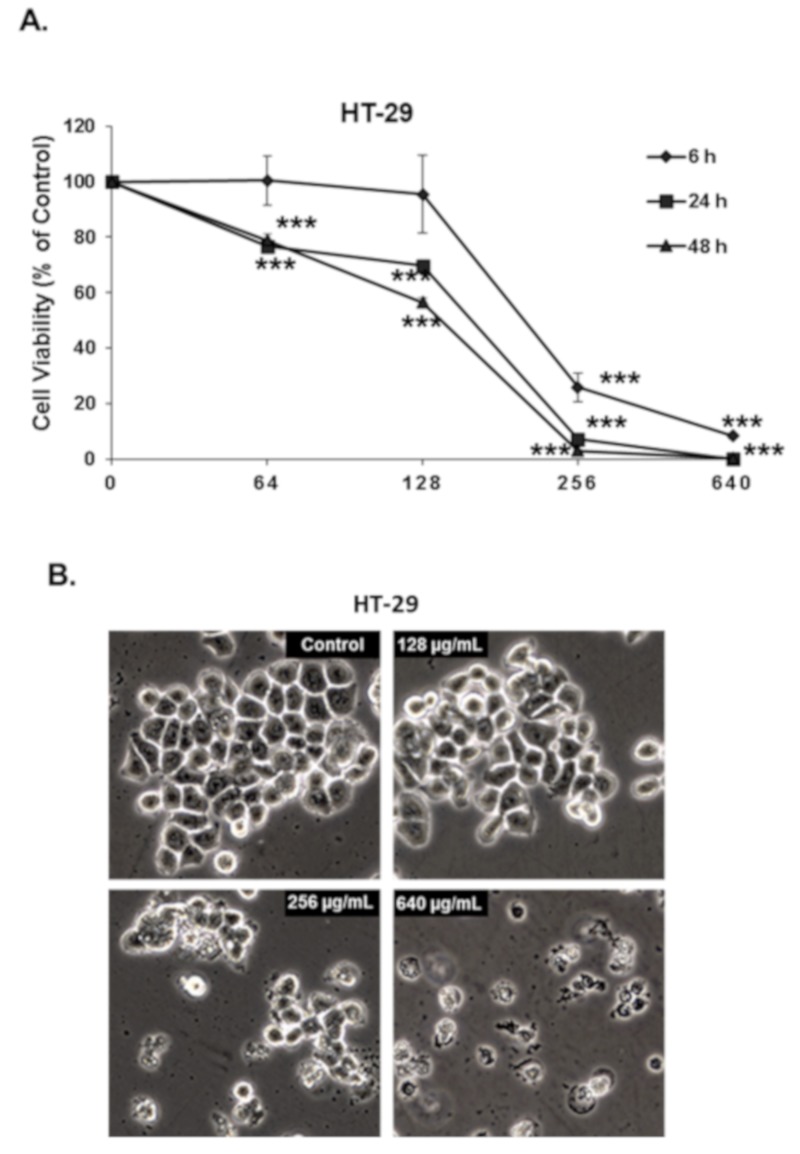
*Origanum majorana* essential oil (OMEO) inhibits the cellular viability of HT-29 cells. (**A**) Exponentially growing HT-29 colon cancer cells were treated with and without the indicated concentrations (0, 64, 128, 256, and 640 μg/mL) of OMEO for 6, 24, and 48 h. Viability was measured as described in Section 2.3 Data represent the mean of six independent experiments carried out in triplicate. Values are represented as mean ± SD of *n* = 4 (*** p < 0.001). (**B**) Morphological changes in OMEO-treated HT-29 cells. Morphological changes observed in the treated HT-29 cells after 6 h of treatment with the indicated concentration of OMEO. Cells were observed under EVOS XL Core Cell Imaging System (Life Technologies) at 400×.

We have tested the effect of increasing concentrations at different times (6, 24, and 48 h) of OMEO on the proliferation of HT-29 colon cancer cells. As shown in Figure 1A, OMEO significantly decreased the cellular viability in a time- and concentration-dependent manner. The IC50 was ~210, 167, and 142 µg/mL at 6, 24, and 48 h, respectively. It is worth mentioning that at 6 h incubation, only a concentration of 256 µg/mL or higher of OMEO caused a significant decrease in cell viability. Microscopy observation of the OMEO-treated revealed morphological changes (smaller and rounded shape) characteristic of dying cells, observable at 256 µg/mL as early as 6 h post-treatment (Figure 1B) and suggesting the rapid killing activity of OMEO at this concentration.

**Figure 2:**
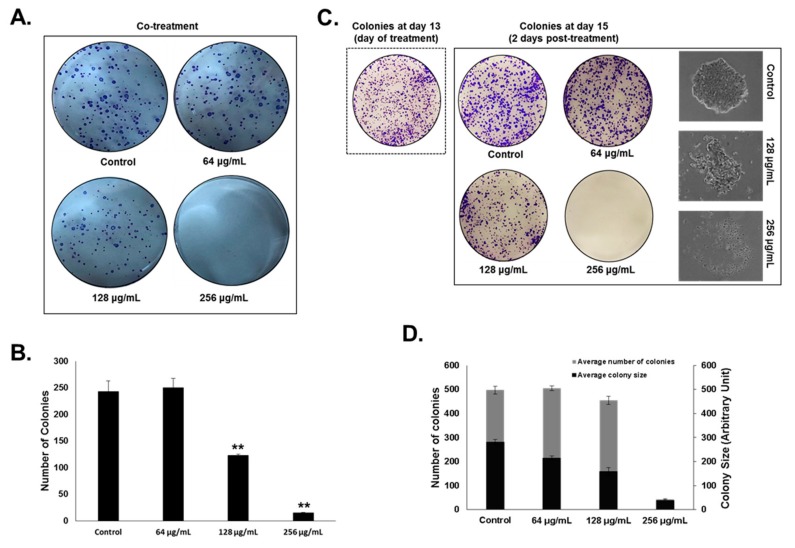
OMEO inhibits HT-29 colony growth. (**A**,**B**) Inhibition of HT-29 colony growth by various concentrations of OMEO (0, 64, 128, and 256 μg/mL) was assessed by measuring the number of the colonies obtained in control and OMEO-treated plate, as described in Section 2.5 Values are represented as mean ± SD of *n* = 3 (** *p* < 0.005). (**C**) HT-29 colonies were first allowed to form in normal media for 13 days as described in Section 2.5 Formed colonies were then treated with or without increasing concentrations of OMEO, and allowed to grow for three more days before crystal violet staining. The size and morphology of the growing colonies was tracked over time with the EVOS XL Core Cell Imaging System (Life Technologies) at 400×. (**D**) Inhibition of colony growth was assessed by measuring the number and size (surface area) of the colonies obtained in control and OMEO-treated plates, as described in Section 2.5 Data represent the mean of three independent experiments carried out in duplicate. Values are represented as mean ± SD of *n* = 3 (* p < 0.05, ** p < 0.005).

Next, we sought to examine the effect of OMEO on HT-29 colony formation. HT-29 cells were treated with and without increasing concentrations of OMEO and cells were allowed to grow for 13 days to form colonies. As shown in Figure 2A,B, OMEO significantly inhibited colony formation by HT-29 cells. A concentration of 256 µg/mL almost completely abolished the ability of HT-29 to form colonies. We have also examined the ability of OMEO to inhibit the growth of already formed HT-29 colonies. Toward that, HT-29 cells were allowed to form visible colonies for 13 days and then treated with and without increasing concentrations of OMEO for three more days. As shown in Figure 2C,D, OMEO caused a significant decrease not only in the number of colonies, but also in the size of the already formed colonies. Microscopic observation of the treated colonies clearly shows a regression in the number of colony-forming cells, clearly indicating massive cell death (Figure 2C).

### 3.3. Origanum majorana Essential Oil Induces Apoptotic Cell Death of Colon Cancer Cells through Activation of the Extrinsic and Intrinsic Pathways

**Figure 3:**
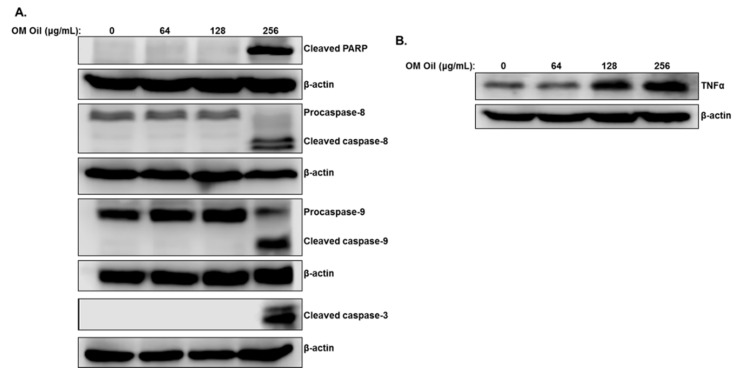
Induction of caspase-8, -9, and -3-mediated apoptosis by OMEO in HT-29 cells. (**A**) Western blot analysis of cleaved PARP, caspase-8, -9, and -3 activation in HT-29 cells treated with increasing concentrations of OMEO (0, 64, 128, and 256 μg/mL) for 6 h. (**B**) Western blot quantification of TNF-α protein in OMEO-treated HT-29 cells.

We have explored the mechanism of OMEO-induced cell death by scoring for markers of apoptotic cell death. Proteins extracted from HT-29 cells treated for 6 h with increasing concentrations of OMEO and markers of apoptosis were examined by Western blot. Figure 3A shows an accumulation of cleaved PARP and activation of the executioner caspase-3 and both initiator caspases-8 and -9 only at a concentration of 256 µg/mL OMEO, in agreement with cellular viability data that showed that a lower concentration of OMEO does not affect the viability of HT-29. This suggests that OMEO triggers the activation of both extrinsic and intrinsic apoptotic pathways in HT-29 colon cancer cells.

The expression of TNF-α, a cytokine involved in the initiation of the extrinsic apoptotic pathway, was also examined and was found to be upregulated in OMEO-treated cells (Figure 3B). This suggests a potential involvement of the TNF-α signaling pathway in triggering the apoptotic cell death program in HT-29 cells.

### 3.4. OMEO Triggers Autophagy through Downregulation of the mTOR/p70S6K Signaling Pathway

**Figure 4:**
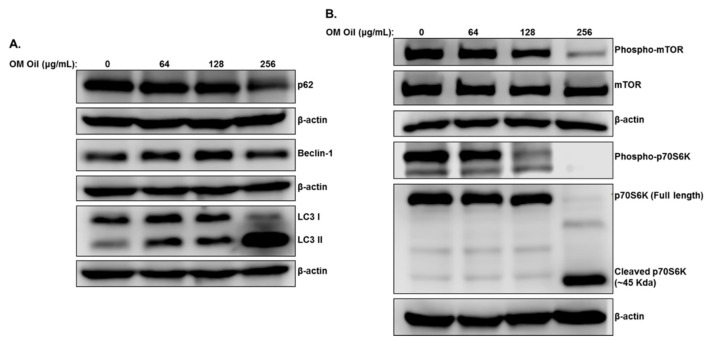
OMEO induces autophagy associated with downregulation of mTOR/p70S6K in HT-29 cells. (**A**) Induction of autophagy by OMEO. Western blotting analysis of marker of autophagy, p62(SQSTM1), Beclin-1, and LC3 II in OMEO-treated HT-29 cells. Cells were treated with or without an increasing concentration of OMEO (0, 64, 128, and 256 μg/mL) for 6 h, then whole cell proteins were extracted and subjected to Western blot analysis, as described in Section 2.6 (**B**) Downregulation of the mTOR/p70S6K by OMEO. Western blot analysis for the phosphorylated and non-phosphorylated form of mTOR and p70S6K.

We have previously shown that *Origanum majorana* ethanolic extract triggers abortive autophagy in colon cancer cells. We, therefore, interrogated whether OMEO also triggers autophagy in colon cancer cells. As shown in Figure 4A, OMEO treatment for 6 h causes an increase in lipidized LC3II, a marker of phagophore formation, and Beclin-1, a marker of autophagosome formation, and a decrease in p62 (SQSTM1), a marker of autophagic flux. Hence, autophagy is triggered in response to OMEO, but unlike the ethanolic extract, the essential oil induces productive autophagy in HT-29 cells.

mTORC1, a serine/threonine kinase, plays a major role in regulating autophagy in colorectal cancer [30]. Stimulation of mTORC1 signaling blocks autophagy, whereas its inhibition triggers this process. Western blot analysis of OMEO-treated HT-29 cells showed a decrease in the level of phosphorylation of mTORC1 at Ser2448, while there was no effect on the total mTORC1. This strongly suggests that inhibition of the mTOR signaling pathway by OMEO accounts for the activation of the autophagy program in HT-29 cells. We next examined the level of phosphorylated p70S6K, a main downstream target of the mTOR pathway involved in many processes, including protein synthesis, cell proliferation, migration and survival. As shown in Figure 4B, OMEO, at concentration of 256 μg/mL, caused a complete inhibition of phosphorylated p70S6K. Strikingly, we also found that OMEO led to depletion of total p70S6K, with a concomitant increase in the immunoreactive band of ~45 KDa. This suggests that downregulation of total p70S6K by OMEO may be a result of proteolytic cleavage of the full-length protein.

### 3.5. Origanum majorana Essential Oil Induces Protective Autophagy and Apoptotic Cell Death

**Figure 5:**
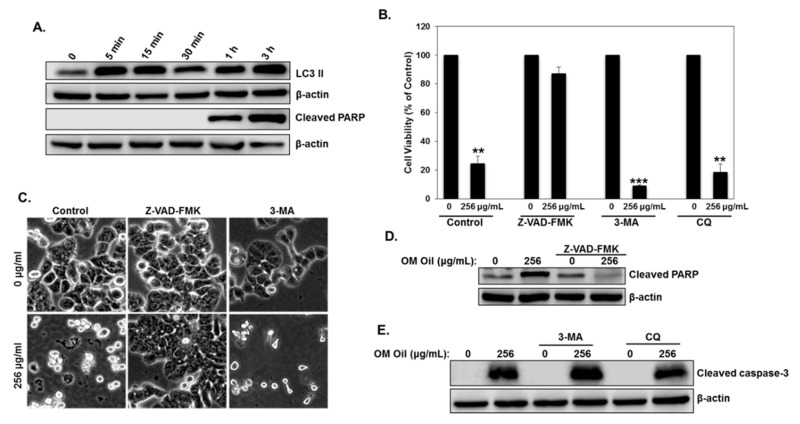
Protective autophagy and apoptotic cell death in HT-29 cells in response to OMEO. (**A**) Time-course analysis of LC3 II and cleaved PARP in OMEO-treated HT-29 cells. Cells were treated with 256 μg/mL OMEO and protein levels were determined by Western blot at different time points (0, 5 min, 15 min, 30 min, 1 h, and 3 h) post-treatment. (**B**) Blockade of autophagy increases cell death, while inhibition of apoptosis promotes cell survival. HT-29 cells were pretreated with Z-VAD-FMK, 3-MA, or CQ and then incubated for 6 h with 256 μg/mL OMEO. Cell viability was determined as described in Section 2.3 (**C**) Micrograph observation of HT-29 cells pretreated with or without pan-caspase inhibitor (Z-VAD-FMK) and 3-MA, as described above. (**D**) Western blotting analysis of cleaved PARP in OMEO cell pretreated with Z-VAD-FMK. (**E**) Western blotting analysis of cleaved caspase-3 with or without 3-MA or CQ.

We have shown that OMEO induces autophagy and activates the apoptotic cell death program in HT-29 colon cancer cells. We first examined the time at which both events occurred. As shown in Figure 5A, autophagy was detectable as early as 5 min post-OMEO treatment and preceded apoptosis, which was detectable only after 1 h.

While there is no doubt about the outcomes of apoptosis in OMEO-treated cells, a question regarding the role of autophagy—cytoprotective or pro-death—remains. To answer this question, the cellular viability of HT-29 cells was measured in the first place in cells pretreated with a pan-caspase-inhibitor (Z-VAD-FMK) or an autophagy inhibitor (CQ and 3-MA) prior to treatment with OMEO. We found that inhibition of apoptosis significantly reduced cell death (Figure 5B). Indeed, cellular viability rose from ~25% in cells treated only with OMEO to ~87% in cells pretreated with Z-VAD-FMK before the addition of OMEO. Microscopy observation confirmed these results. As shown in Figure 5C, OMEO alone caused massive cell death (left panel); Z-VAD-FMK pretreated cells, on the other hand, were indistinguishable from control cells (middle panel). Inhibition of apoptosis by Z-VAD-FMK was confirmed by the absence of cleaved PARP in Z-VAD-FMK pretreated cells (Figure 5D). Interestingly, the inhibition of early stage autophagosome formation by 3-MA or late-stage autophagy (autophagolysosome formation) by CQ caused more apoptotic cell death than cells treated with OMEO alone (Figure 5B,C,E). Cellular viability significantly dropped from ~25% in cells treated with OMEO only to ~8% and 18% in cells pretreated with 3-MA and CQ, respectively. Hence, these results suggest that OMEO-induced autophagy plays a cytoprotective role in HT-29 cells.

### 3.6. OMEO Induces p38 MAPK-Mediated Protective Autophagy and Apoptosis

**Figure 6:**
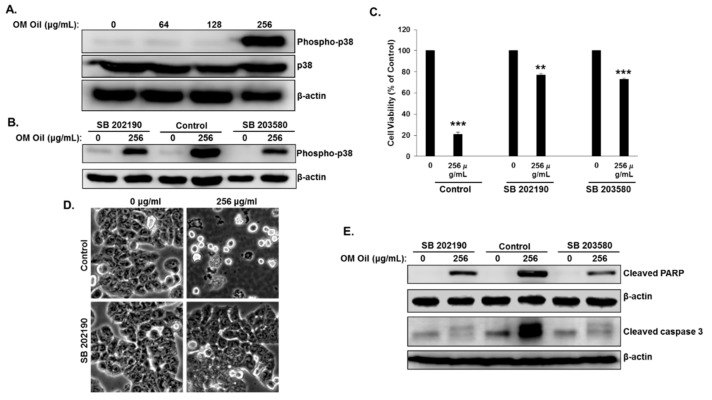
p38 MAPK-dependent activation of apoptosis in OMEO-treated cells. (**A**) OMEO activates p38 MAPK in HT-29 cells. Western blotting analysis of phosphorylated and total p38 in OMEO-treated HT-29 cells. Cells were treated with or without an increasing concentration (0, 64, 128, and 256 μg/mL) for 6 h, then whole cell proteins were subjected to Western blot analysis. (**B**) Western blotting analysis of phosphorylated p38 in the presence of p38 inhibitors (SB 202190 and SB 203580). (**C**) Inhibition of p38 MAPK abrogates the OMEO-induced apoptotic cell death. HT-29 cells were pretreated with p38 inhibitors and cellular viability was determined as described in Section 2.3 (**D**) Morphological changes observed in the treated HT-29 pretreated with p38 inhibitors prior the incubation with OMEO. Cells were observed under EVOS XL Core Cell Imaging System (Life Technologies) at 400×. (**E**) Western blotting analysis of cleaved PARP and cleaved caspase-3 in cell pretreated with and without SB 202190 and SB 203580.

An increasing number of studies have reported the involvement of the MAPK signaling pathway in the regulation of autophagy and apoptosis in response to anticancer drugs. We examined the effect of OMEO on the p38 MAPK signaling pathway in HT-29 colon cancer cells. Toward this, HT-29 cells were treated with increasing concentrations of OMEO for 6 h and we examined the levels of the phosphorylated form of p38 by Western blotting. We found that OMEO dramatically increased the level of phosphorylated p38 only when used at 256 µg/mL, a concentration at which apoptosis is detected (Figure 6A). No activation of the p38 pathway was detected at the lower concentration, at which apoptosis was also absent. It is worth mentioning that a strong activation of the p38 MAPK pathway was reported to lead cells into apoptosis [31]. Hence, it is tempting to hypothesize that the activation of p38 MAPK by OMEO contributes to the activation of apoptotic cell death in HT-29 cells. To test this hypothesis, we inhibited the activation of this pathway by two p38 MAPK inhibitors (SB 202190 and SB 203580) and examined their effects on cellular viability. The inhibition of p38 activation was confirmed by Western blot analysis. As shown in Figure 6B, both inhibitors dramatically reduced the level of phosphorylated p38. We found that both p38 inhibitors significantly rescued HT-29 from cell death (Figure 6C). Cell viability rose from ~25% in cells treated only with OMEO to ~76% and 72% in cells pretreated with SB 202190 and SB 203580, respectively. In agreement with the cell viability data, microscopy observations show an absence of cell death in SB-202190-treated cells (Figure 6D). Inhibition of apoptosis in SB 202190- and SB 203580-pretreated HT-29 cells was confirmed by Western blot. As shown in Figure 6E, both p38 inhibitors blocked caspase 3 activation and PARP cleavage. Altogether, these results suggest that OMEO induces p38 MAPK-dependent apoptosis.

**Figure 7:**
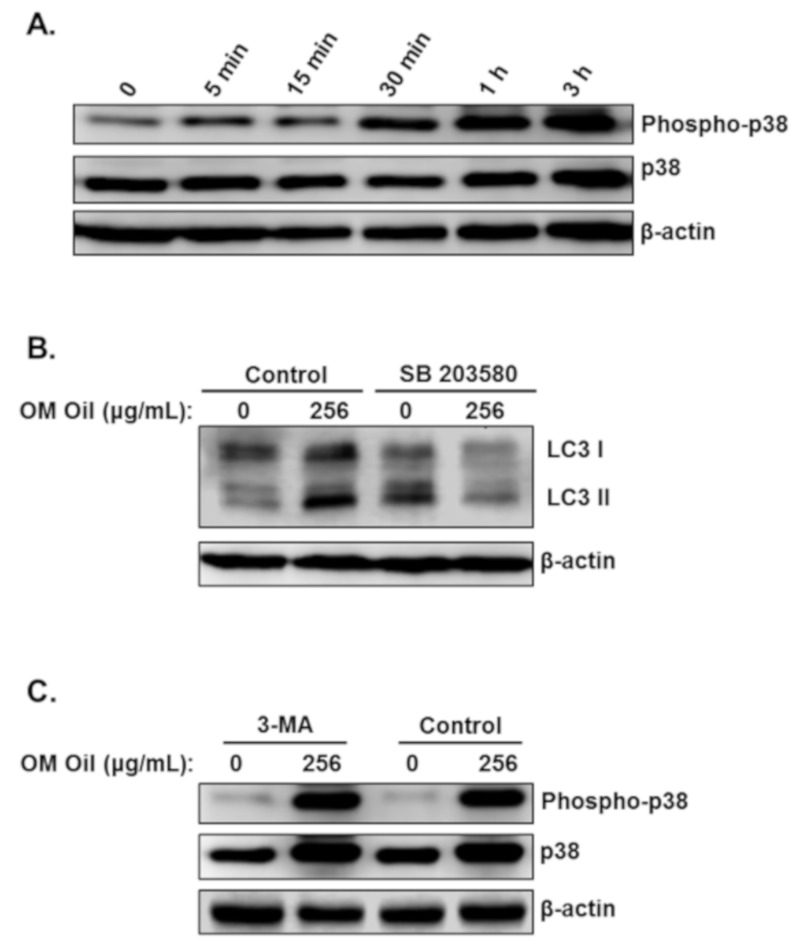
p38 MAPK-mediated protective autophagy. (**A**) Time-course measurement of phosphorylated and total p38 level in OMEO-treated cells. Cells were treated with 256 μg/mL OMEO and the level of active and total p38 was examined at different time points (0, 5 min, 15 min, 30 min, 1 h and 3 h). (**B**) LC3 I and LC3 II levels were measured in cells pretreated with SB 203580. (**C**) Western blotting analysis of phosphorylated and total p38 in cells pretreated with and without 3-MA.

Next, we examined the link between the activation of the p38 MAPK pathway and induction of autophagy. We first examined the time at which activation of p38 MAPK occurred. We found that the activation of p38 MAPK was detectable as early as 5 min and maximal activation was detected at 3 h post-OMEO treatment (Figure 7A). Interestingly, we found that apoptosis was also triggered at the same time, i.e., 5 min post-OMEO treatment (Figure 5A), suggesting that activation of p38MAPK and autophagy occurred simultaneously in HT-29 cells. To investigate whether activation of the p38 MAPK affects OMEO-induced autophagy, HT-29 cells were pretreated with SB203580 before treatment with 256 μg/mL OMEO, and autophagy was scored by examining the level of the autophagy marker, lipidized LC3 II. As shown in Figure 7B, blockade of p38 MAPK attenuated the level of autophagy, as deduced by the reduction of LC3 II. Blockade of autophagy by 3-MA caused no changes in the level of phosphorylated p38 (Figure 7C). Altogether, the activation of p38 MAPK is closely associated with OMEO-induced autophagy and apoptosis in HT-29 colon cancer cells.

### 3.7. OMEO Induces p38 MAPK-Mediated Caspase-Dependent Cleavage of p70S6K.

**Figure 8:**
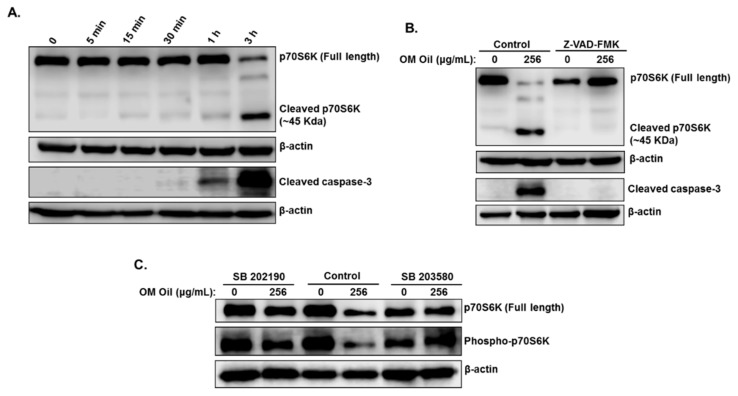
p38 MAPK-dependent caspase-dependent cleavage of p70S6K. (**A**) Time course measurement of p70S6K and cleaved caspase-3 in OMEO-treated HT-29 cells. Cells were treated with 256 μg/mL OMEO and protein levels were examined at different time points (0, 5 min, 15 min, 30 min, 1 h, and 3 h). (**B**) Western blotting analysis of p70S6K and cleaved caspase-3 in cells pretreated with Z-VAD-FMK. (**C**) Western blot analysis of full-length and phosphorylated p70S6K in cells pretreated with SB 202190 and SB 203580.

In addition to its role in cell proliferation, motility, and survival, the p70S6K protein was shown to be implicated in the inhibition of apoptosis in cancer cells. The downregulation of this protein by caspase-3-dependent cleavage was shown to contribute to elicit the activation of apoptosis in these cancer cells [32]. To elucidate the mechanism by which total p70S6K was downregulated in OMEO, we first examined the order in which p70S6K cleavage and caspase 3 activation occurred in HT-29 cells. Toward this, a time course examination of protein levels was performed. We found that the cleavage of p70S6K was slightly detectable after 1 h, but the cleavage was almost complete 3 h post-treatment (Figure 8A). Active caspase-3 was also detectable at 1 h and more abundant after 3 h of treatment (Figure 8A).

To incriminate apoptosis in the cleavage of p70S6K, we examined the effect of caspase inhibition by Z-VAD-FMKZ-VAD on the level and integrity of p70S6K. As shown in Figure 8B, inhibition of caspases completely restored the level of full-length p70S6K protein and blocked the generation of the 45-kDa fragment, a cleaved form of p70S6K. Blockade of caspase 3 activation was confirmed by Western blotting (Figure 8B). Our data demonstrate that OMEO promotes caspase-dependent cleavage of p70S6K.

Having shown that p70S6K undergoes caspase-dependent cleavage and that apoptosis activation in p38-MAPK-dependent, we are tempted to suggest that p38 MAPK activation serves as a trigger for p70S6K cleavage. To test this hypothesis, the level and integrity of p70S6K were examined in HT-29 cells pretreated with the two p38 inhibitors, SB 202190 and SB 203580. As shown in Figure 8C, both inhibitors significantly rescued p70S6K from cleavage. The level of full-length p70S6K in cells treated with p38 inhibitors and OMEO is comparable to the level of this protein in cells treated with only the p38 inhibitors. Interestingly, both p38 inhibitors restored the level of phosphorylated p70S6K (Figure 8C), activated by the mTOR pathway, suggesting that autophagy is also reduced when p38 MAPK is inhibited. This result is in agreement with our results in Figure 7B, which shows a decrease in the level of LC3 II upon blockade of p38 MAPK activation. In conclusion, our data suggest that p38-mediated caspase-dependent cleavage of p70S6K might contribute to enhance the level apoptosis in HT-29 cells.

### 3.8. DNA Damage-Independent Activation of p38 MAPK

**Figure 9:**
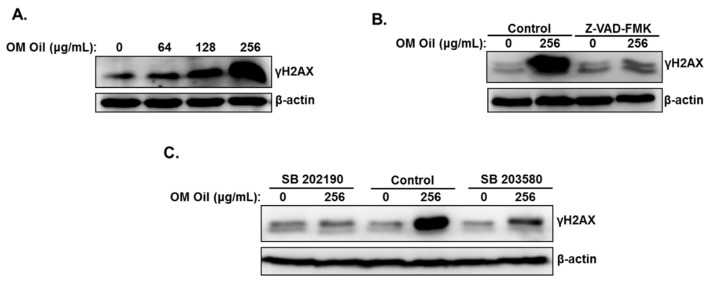
DNA damage in OMEO-treated HT-29 cells. (**A**) Accumulation of γH2AX, a marker of DNA damage, in OMEO-treated cells. HT-29 cells were treated with and without increasing concentrations of OMEO for 6 h and DNA damage was analyzed by Western blot, by determining the level of γH2AX accumulation. (**B**) Western blotting analysis of γH2AX in cells pretreated with and without Z-VAD-FMK. (**C**) Western blotting analysis of γH2AX in cells pretreated with and without SB 202190 and SB 203580.

Studies reported that p38 MAPK signaling pathway could be activated by DNA damage and mediate apoptotic cell death [33]. We, therefore, decided to investigate whether OMEO induces DNA damage in HT-29 cells. We found that OMEO increased the levels of γH2AX (Figure 9A), indicative of double-strand breaks. To test whether DNA damage is a cause or a consequence of p38 MAPK activation, the level of γH2AX was measured in cells treated with the p38 inhibitors prior to incubation with OMEO. Inhibition of p38 MAPK totally abolished the DNA damage induced by OMEO (Figure 9B). Similarly, we found that DNA damage did not occur in cells incubated with the inhibitor of apoptosis prior to treatment with OMEO (Figure 9C). Based on these results, we can conclude that activation of p38 MAPK in colon cancer by OMEO occurs independently of DNA damage through a mechanism yet to be uncovered.

## 4. Discussion

A large body of recent literature reported the ability of essential oils to exert an anticancer effect though their ability to affect various pathways and cellular mechanisms. Hence, the use of essential oils as a source of potent anticancer compounds has gained a great deal of interest in the field of cancer therapy in recent years. We and others have previously extensively documented the anticancer activities of *Origanum majorana* ethanolic extract against various types of cancer including breast [17,18], colon [19], and liver [34]. However, to the best of our knowledge, no literature data on the anticancer activity of *Origanum majorana* essential oil are available. This prompted us to investigate the anti-colon cancer activity of OMEO. Our data showed for the first time that OMEO significantly inhibited the proliferation of colon cancer cells, induced cytoprotective autophagy, and activated the intrinsic and extrinsic apoptotic pathway through inhibition of the mTOR/p70S6K and activation of the p38 MAPK pathway. Intriguingly, we found that p38 MAPK induces both autophagy and apoptosis and mediates a caspase-dependent cleavage of p70S6K. To the best of our knowledge, this study is the first to report a link between p38 signaling pathway and cleavage of p70S6K.

It is not surprising that essential oils and alcoholic extracts possess different biological activities. This could be due to differences in the composition and/or concentration of phytochemicals present in the EO versus alcoholic extract. The essential oil and ethanolic extract of two plants, namely *Ocimum basilicum* and *Thymus algeriensis*, were shown to exhibit different anticancer activities against various cancer cell lines. The EO of the two plants showed a much greater antiproliferative activity than the EE [35]. We have recently reported that *Origanum majorana* ethanolic extract induced abortive autophagy and apoptosis in HT-29 cells. Intriguingly, we found that autophagy represented the major mechanism of cell death, induced while apoptosis was minimal [19]. This suggests that OMEO and OMEE exert anti-colon cancer effects through different mechanisms, with autophagy playing a pro-death role in response to OMEE and a pro-survival role in response to OMEO. Such differences in the biological activities can be imputed to variations in the chemical composition between OMEO and OMEE [36,37]. It has been reported that Terpinen-4-ol is the main component of the extract and the oil; however, its concentration in the essential oil was almost twice as high as in the OMEE. Additionally, Linalool, c-Terpinene, and p-Cymol are present in high amounts in EO compared to EE. Moreover, several compounds, including Camphene, b-Myrcene, and Terpenyl-acetate, have been detected in the essential oil, while they are absent in EE [36].

Although the role of apoptosis in cell death in unquestionable, autophagy, however triggered in response to anticancer therapy, might lead to opposite outcomes. It can be associated with cell death, leading to the activation of the pro-death signaling pathway in cancer cells [34]. In other circumstances, autophagy can be cytoprotective, promoting cell survival, resistance to anticancer treatments, and protection from apoptosis [38,39]. In this latter case, inhibition of autophagy was shown to increase cell death in various cancer cell types in response to anticancer drugs, whether synthetic or of natural origin [39,40]. Here we showed that OMEO induced a cytoprotective autophagy and apoptosis in HT-29 cells. However, autophagy, detected as early as 5 min post-OMEO treatment, preceded apoptosis activation, which was maximal after 3 h treatment. We showed that the induction of autophagy is mediated by the inhibition of the autophagy regulator, the mTOR/p70S6K signaling pathway. Interestingly, pharmacological blockade of autophagy by 3-MA and CQ, autophagy inhibitors of autophagosome and autophagolysosome formation, potentiated the proapoptotic activity of OMEO in HT-29 cells. In addition, we showed that inhibition of apoptosis by the pan-caspase inhibitor Z-VAD-FMK alleviated OMEO-induced apoptotic cell death. These findings strongly suggest that autophagy, triggered by OMEO, plays a protective role and that apoptosis represents the main mechanism of cell death.

Whether autophagy elicits or inhibits apoptosis seems to depend on the cell type and the nature and duration of the stimulus/stress [41]. Autophagy directly or indirectly limits or delays the onset of apoptosis—for example, by eliminating cytotoxic protein aggregates and damaged organelles [42]. Still, the exact mechanism through which the switch from autophagy to apoptosis occurs remains poorly understood. The protein p38 MAPK was shown to regulate both processes in different ways. Indeed, studies reported that p38 suppresses autophagy but promotes apoptosis, as is the case in selenite-treated leukemia cells [43]. Other studies showed that p38 MAPK could trigger both autophagy and apoptosis in response to anticancer drugs. Resveratrol, a naturally occurring phytoalexin, was shown to mediate its anticancer activities in T-cell Acute Lymphoblastic Leukemia, thorough simultaneous activation of protective autophagy and apoptosis. The mechanism of action of Resveratrol involves the inhibition of AKT/mTOR/p70S6K/4E-BP1 and the activation of p38-MAPK signaling pathways [39]. Pharmacological inhibition of autophagy by 3-MA increased Resveratrol-induced cell death, while inhibition of p38 MAPK by SB 203,580 exerted the opposite effect [39]. Also, Salinomycin, a natural compound isolated from *Streptomyces albus*, was shown to induce p38 MAPK-mediated downregulation of the AKT/mTOR pathway, protective autophagy, and apoptosis in human prostate cancer cells [44]. Inhibition of autophagy with 3-MA enhanced Salinomycine-induced apoptosis and pretreatment with the p38 inhibitor SB 203,580 decreased salinomycin-induced autophagy [44]. Here we showed that OMEO activated both autophagy and apoptosis, with autophagy occurring first. Interestingly, we showed that activation of autophagy and apoptosis seems to depend on p38MAK activation. Indeed, we showed that inhibition of p38 MAPK by the two p38 inhibitors (SB203580 and SB 202190) not only alleviated apoptosis cell death but also reduced the level of autophagy, as shown by a decrease in the level of lipidized LC3II and rescue of phosphorylated p70S6K. These results strongly suggest that OMEO activates p38 MAPK, which then serves as a trigger to induce autophagy and apoptosis in HT-29 cells. The mechanism by which p38 MAPK switches from autophagy to apoptosis could be explained by the extent of p38 MAPK activation. A short exposure of HT-29 cells to OMEO triggers a moderate activation of p38 MAPK, which, in this case, favors autophagy induction. However, as damage to the cell increases due to longer exposure to OMEO, the level of p38 activation also increases, causing cells to activate the apoptotic cell death program. In agreement with this, a strong activation of the p38 MAPK pathway was reported to lead cells into apoptosis [31].

p70S6 kinase (p70S6K), a serine/threonine protein kinase, phosphorylated through the PI3K/mTOR pathway, was reported to play a role in cellular proliferation, survival, motility metastasis, and chemotherapy drug resistance in cancer cells [45]. Upregulation of p70S6K activity, through overexpression, was reported in several cancer tissues. The overexpression of p70S6K signaling might contribute to the growth, invasion, and metastasis of colon cancer and, thus, it is considered a potential marker to indicate the aggressive behaviors and prognosis of human colorectal carcinomas [46]. Overexpression of p70S6K was also reported in hepatocellular carcinoma (HCC) and was incriminated in the progression of HCC, and thus should be considered as a potential molecular target for HCC therapy [47]. In agreement with these previous findings, in vitro overexpression of p70S6K was also shown to promote cellular proliferation and inhibit apoptosis in breast cancer [48]. Conversely, RNA interference-mediated knockdown of p70S6K decreased the cellular proliferation of human glioblastoma cell lines U251 [49] and sensitized resistant colon cancer cells to selumetinib [50]. Dhar et al. showed that cisplatin caused a caspase-3-dependent cleavage of p70S6K and that proteolytic cleavage is important for cisplatin-induced apoptosis in H69 and A549 colon cancer cells [32]. Also, the flavone 3,3′-diamino-4′-methoxyflavone (DD1) inhibited cellular proliferation and induced apoptotic cell death in several acute myeloid leukemia cell lines associated with caspase proteolytic cleavage of p70S6K [51]. The exact molecular mechanism through which these anticancer drugs specifically targets p70S6K to caspase-dependent cleavage remain unknown. Here we showed that OMEO induced a caspase-dependent cleavage of p76S6K. Interestingly, we found that targeting of p70S6K to caspase-dependent cleavage was mediated by the activation of p38 MAPK. Inhibition of apoptosis by pan-caspase inhibitors and pharmacological blockade of p38 MAPK activation by p38 inhibitors prevented p70S6K. It is worth mentioning that this study is the first to implicate p38 MAPK in targeting p70S6K to caspase-dependent cleavage. We hypothesize that cleavage of p70S6K might sensitize HT-29 cells to undergo massive apoptosis. Our findings, along with previous findings, highlight the importance of p70S6K in human cancer and provide a strong rationale for the development of inhibitors targeting this protein as anticancer drugs.

In conclusion, our study demonstrated that OMEO exerts anti-colon cancer activity through activation of p38 MAPK signaling, induction of protective autophagy, associated with downregulation of the mTOR/p70S6K pathway, and activation of the extrinsic and intrinsic apoptotic pathway. Induction of autophagy and apoptosis was mediated through a p38-MAPK-dependent mechanism. Interestingly, p38 MAPK was found to target p70S6K to caspase cleavage. In light of these findings, in future studies, it would be interesting to explore whether a combination therapy of currently clinically used anticancer therapeutics with OMEO in drug-resistant colorectal cancer cells would sensitize resistant cells to undergo cell death.

## Supplementary Materials

The following are available online at https://www.mdpi.com/2218-273X/10/3/412/s1, Figure S1: Commercial *Origanum majorana* essential oil brand used in this study.; Figure S2: Chromatograms (A) and names and mass of compounds (B) identified in OMEO by HPLC-MS.

## References

[1] Kuipers E.J., Grady W.M., Lieberman D., Seufferlein T., Sung J.J., Boelens P.G., van de Velde C.J.H., Watanabe T. (2015). Colorectal cancer. Nat. Rev. Dis. Primers.

[2] Peluso G., Incollingo P., Calogero A., Tammaro V., Rupealta N., Chiacchio G., Sandoval Sotelo M.L., Minieri G., Pisani A., Riccio E. (2017). Current Tissue Molecular Markers in Colorectal Cancer: A Literature Review. Biomed. Res. Int..

[3] Newman D.J., Cragg G.M. (2016). Natural Products as Sources of New Drugs from 1981 to 2014. J. Nat. Prod..

[4] Balunas M.J., Kinghorn A.D. (2005). Drug discovery from medicinal plants. Life Sci..

[5] Nobili S., Lippi D., Witort E., Donnini M., Bausi L., Mini E., Capaccioli S. (2009). Natural compounds for cancer treatment and prevention. Pharmacol. Res. Off. J. Ital. Pharmacol. Soc..

[6] Petrovska B. (2012). Historical review of medicinal plants’ usage. Pharmacogn. Rev..

[7] Jain S., Dwivedi J., Jain P.K., Satpathy S., Patra A. (2015). Medicinal Plants for Treatment of Cancer: A Brief Review. Pharmacogn. J..

[8] Raut J.S., Karuppayil S.M. (2014). A status review on the medicinal properties of essential oils. Ind. Crop. Prod..

[9] Adorjan B., Buchbauer G. (2010). Biological properties of essential oils: An updated review. Flavour Fragr. J..

[10] Tasdemir D., Kaiser M., Demirci B., Demirci F., Baser K.H.C. (2019). Antiprotozoal Activity of Turkish *Origanum onites* Essential Oil and Its Components. Molecules.

[11] Rajkumar S., Jebanesan A. (2008). Repellent activity of selected plant essential oils the malarial fever mosquito Anopheles stephensi. Trop. Biomed..

[12] Goel P., Vasudeva N. (2015). Origanum majorana L. -Phyto-pharmacological review. J. Essent. Oil Bear. Plants.

[13] Duletic S., Alimpić Aradski A., Kolarevic S., Vuković-Gačić B., Oaldje M., Živković J., Šavikin K., Marin P. (2018). Antineurodegenerative, antioxidant and antibacterial activities and phenolic components of Origanum majorana L. (Lamiaceae) extracts. J. Appl. Bot. Food Qual..

[14] Leeja L., Thoppil J. (2007). Antimicrobial activity of methanol extract of Origanum majorana L. (Sweet marjoram). J. Environ. Biol. Acad. Environ. Biol. India.

[15] Amor G., Caputo L., La Storia A., De Feo V., Mauriello G., Fechtali T. (2019). Chemical Composition and Antimicrobial Activity of *Artemisia herba-alba* and *Origanum majorana* Essential Oils from Morocco. Molecules.

[16] Della Pepa T., Elshafie H.S., Capasso R., De Feo V., Camele I., Nazzaro F., Scognamiglio M.R., Caputo L. (2019). Antimicrobial and Phytotoxic Activity of *Origanum heracleoticum* and *O. majorana* Essential Oils Growing in Cilento (Southern Italy). Molecules.

[17] Al Dhaheri Y., Attoub S., Arafat K., Abuqamar S., Viallet J., Saleh A., Al Agha H., Eid A., Iratni R. (2013). Anti-metastatic and anti-tumor growth effects of Origanum majorana on highly metastatic human breast cancer cells: Inhibition of NFκB signaling and reduction of nitric oxide production. PLoS ONE.

[18] Dhaheri Y., Eid A., AbuQamar S., Attoub S., Khasawneh M., Aiche G., Hisaindee S., Iratni R. (2013). Mitotic Arrest and Apoptosis in Breast Cancer Cells Induced by Origanum majorana Extract: Upregulation of TNF-α and Downregulation of Survivin and Mutant p53. PLoS ONE.

[19] Benhalilou N., Alsamri H., Alneyadi A., Athamneh K., Alrashedi A., Altamimi N., Al Dhaheri Y., Eid A.H., Iratni R. (2019). Origanum majorana Ethanolic Extract Promotes Colorectal Cancer Cell Death by Triggering Abortive Autophagy and Activation of the Extrinsic Apoptotic Pathway. Front Oncol..

[20] Yazdanparast R., Shahriyary L. (2008). Comparative effects of Artemisia dracunculus, Satureja hortensis and Origanum majorana on inhibition of blood platelet adhesion, aggregation and secretion. Vasc. Pharmacol..

[21] Soliman A.M., Desouky S., Marzouk M., Sayed A.A. (2016). Origanum majorana Attenuates Nephrotoxicity of Cisplatin Anticancer Drug through Ameliorating Oxidative Stress. Nutrients.

[22] Al-Howiriny T., Alsheikh A., Alqasoumi S., Al-Yahya M., ElTahir K., Rafatullah S. (2009). Protective Effect of Origanum majorana L. “Marjoram” on Various Models of Gastric Mucosal Injury in Rats. Am. J. Chin. Med..

[23] Makrane H., Aziz M., Berrabah M., Mekhfi H., Ziyyat A., Bnouham M., Legssyer A., Elombo F.K., Gressier B., Eto B. (2019). Myorelaxant Activity of essential oil from Origanum majorana L. on rat and rabbit. J. Ethnopharmacol..

[24] Mossa A.-T.H., Refaie A.A., Ramadan A., Bouajila J. (2013). Amelioration of Prallethrin-Induced Oxidative Stress and Hepatotoxicity in Rat by the Administration of Origanum majorana Essential Oil. Biomed. Res. Int..

[25] Dantas A.D.S., Klein-Júnior L.C., Machado M.S., Guecheva T.N., Santos L.D.D., Zanette R.A., Mello F.B.D., Pêgas Henriques J.A., Mello J.R.B.D. (2016). Origanum majorana Essential Oil Lacks Mutagenic Activity in the Salmonella/Microsome and Micronucleus Assays. Sci. World J..

[26] Nurzyńska-Wierdak R., Zawiślak G., Kowalski R. (2015). The Content and Composition of Essential Oil of Origanum majorana L. Grown in Poland Depending on Harvest Tme and Method of Raw Material Preparation. J. Essent. Oil Bear. Plants.

[27] Ramos S., Rojas L., Lucena M., Meccia G., Alfredo U. (2011). Chemical Composition and Antibacterial Activity of Origanum majorana L. Essential Oil from the Venezuelan Andes. J. Essent. Oil Res. J. Essent Oil Res.

[28] Vági E., Simándi B., Suhajda Á., Héthelyi É. (2005). Essential oil composition and antimicrobial activity of Origanum majorana L. extracts obtained with ethyl alcohol and supercritical carbon dioxide. Food Res. Int..

[29] Tabanca N., Özek T., Baser K.H.C., Tümen G. (2004). Comparison of the Essential Oils of Origanum majorana L. and Origanum x majoricum Cambess. J. Essent. Oil Res..

[30] Gulhati P., Cai Q., Li J., Liu J., Rychahou P.G., Qiu S., Lee E.Y., Silva S.R., Bowen K.A., Gao T. (2009). Targeted inhibition of mammalian target of rapamycin signaling inhibits tumorigenesis of colorectal cancer. Clin. Cancer Res..

[31] Dolado I., Nebreda A. (2007). Regulation of Tumorigenesis by p38α MAP Kinase. Stress Activated Protein Kinases Top Curr Genet.

[32] Dhar R., Persaud S.D., Mireles J.R., Basu A. (2009). Proteolytic cleavage of p70 ribosomal S6 kinase by caspase-3 during DNA damage-induced apoptosis. Biochemistry.

[33] Han J., Sun P. (2007). The pathways to tumor suppression via route p38. Trends Biochem. Sci..

[34] Fathy S.A., Emam M., Agwa S., Zahra F.A., Youssef F., Sami R.M. (2016). The antiproliferative effect of Origanum majorana on human hepatocarcinoma cell line: Suppression of NF-κB. Cell. Mol. Biol. (Noisy-Le-Grandfrance).

[35] Rezzoug M., Bakchiche B., Gherib A., Roberta A., FlaminiGuido, Kilinçarslan Ö., Mammadov R., Bardaweel S.K. (2019). Chemical composition and bioactivity of essential oils and Ethanolic extracts of Ocimum basilicum L. and Thymus algeriensis Boiss. & Reut. from the Algerian Saharan Atlas. BMC Complement. Altern. Med..

[36] Kawase K., Mothé C., Furtado F., Coelho G. (2013). Changes in essential oil of Origanum vulgare L. affected by different extraction methods. Int. J. Recent Res. Appl. Stud..

[37] Eisenberg-Lerner A., Bialik S., Simon H.-U., Kimchi A. (2009). Life and death partners: Apoptosis, autophagy and the cross-talk between them. Cell Death Differ..

[38] Xiao X., Wang W., Li Y., Yang D., Li X., Shen C., Liu Y., Ke X., Guo S., Guo Z. (2018). HSP90AA1-mediated autophagy promotes drug resistance in osteosarcoma. J. Exp. Clin. Cancer Res..

[39] Liu M., Zhao G., Zhang D., An W., Lai H., Li X., Cao S., Lin X. (2018). Active fraction of clove induces apoptosis via PI3K/Akt/mTOR-mediated autophagy in human colorectal cancer HCT-116 cells. Int. J. Oncol..

[40] Ge J., Liu Y., Li Q., Guo X., Gu L., Ma Z.G., Zhu Y.P. (2013). Resveratrol Induces Apoptosis and Autophagy in T-cell Acute Lymphoblastic Leukemia Cells by Inhibiting Akt/mTOR and Activating p38-MAPK. Biomed. Environ. Sci..

[41] Benbrook D., Long A. (2012). Integration of autophagy, proteasomal degradation, unfolded protein response and apoptosis. Exp. Oncol..

[42] Rubinstein A.D., Kimchi A. (2012). Life in the balance—A mechanistic view of the crosstalk between autophagy and apoptosis. J. Cell Sci..

[43] Jiang Q., Li F., Shi K., Wu P., An J., Yang Y., Xu C. (2014). Involvement of p38 in signal switching from autophagy to apoptosis via the PERK/eIF2??/ATF4 axis in selenite-treated NB4 cells. Cell Death Dis..

[44] Kim K.-Y., Park K.-I., Kim S.-H., Yu S.-N., Park S.-G., Kim Y., Seo Y.-K., Ma J.-Y., Ahn S.C. (2017). Inhibition of Autophagy Promotes Salinomycin-Induced Apoptosis via Reactive Oxygen Species-Mediated PI3K/AKT/mTOR and ERK/p38 MAPK-Dependent Signaling in Human Prostate Cancer Cells. Int. J. Mol. Sci..

[45] Fenton T.R., Gout I.T. (2011). Functions and regulation of the 70kDa ribosomal S6 kinases. Int. J. Biochem. Cell Biol..

[46] Lu Q., Wang J., Yu G., Guo T., Hu C., Ren P. (2015). Expression and clinical significance of mammalian target of rapamycin/P70 ribosomal protein S6 kinase signaling pathway in human colorectal carcinoma tissue. Oncol. Lett..

[47] Li P.D., Zhang W.J., Zhang M.Y., Yuan L.J., Cha Y.L., Ying X.F., Wu G., Wang H.Y. (2012). Overexpression of RPS6KB1 predicts worse prognosis in primary HCC patients. Med. Oncol..

[48] Yamnik R.L., Digilova A., Davis D.C., Brodt Z.N., Murphy C.J., Holz M.K. (2009). S6 Kinase 1 Regulates Estrogen Receptor α in Control of Breast Cancer Cell Proliferation. J. Biol. Chem..

[49] Nakamura J.L., Garcia E., Pieper R.O. (2008). S6K1 plays a key role in glial transformation. Cancer Res..

[50] Grasso S., Tristante E., Saceda M., Carbonell P., Mayor-López L., Carballo-Santana M., Carrasco-García E., Rocamora-Reverte L., García-Morales P., Carballo F. (2014). Resistance to Selumetinib (AZD6244) in colorectal cancer cell lines is mediated by p70S6K and RPS6 activation. Neoplasia.

[51] Piedfer M., Bouchet S., Tang R., Billard C., Dauzonne D., Bauvois B. (2013). p70S6 kinase is a target of the novel proteasome inhibitor 3,3′-diamino-4′-methoxyflavone during apoptosis in human myeloid tumor cells. Biochim. Et Biophys. Acta (Bba) Mol. Cell Res..

