# Supplementary material for: Origanum majorana Essential Oil Triggers p38 MAPK-Mediated Protective Autophagy, Apoptosis, and Caspase-Dependent Cleavage of P70S6K in Colorectal Cancer Cells
Source: Biomolecules. 2020 Mar 6;10(3):412. doi: 10.3390/biom10030412 (PMC7175132; doi:10.3390/biom10030412)
Supplement: Supplementary file 1 [file biomolecules-10-00412-s001.pdf]

Supplementary material

Origanum majorana **Essential Oil triggers a p38 MAPK–mediated protective autophagy, apoptosis and caspase-dependent cleavage of p70S6K in colorectal cancer cells**

Khawlah Athamneh[1q], Aysha Alneyadi[1q], Halima Alsamri[1q], Asma Alrashedi[1], Abdulrasheed Palakott[1], Khaled A. El-Tarabily[1], Ali Eid[2], Yusra Al Dhaheri[1] and Rabah Iratni[1*]

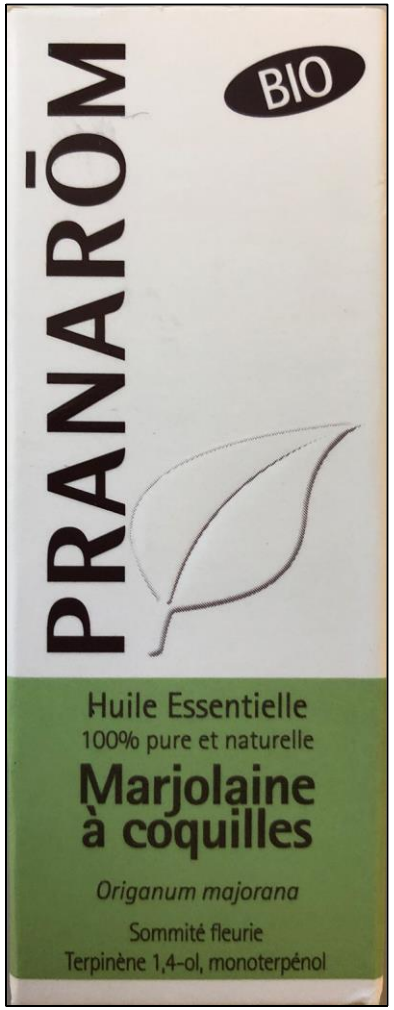

Supplementary Figure 1. Commercial *Origanum majorana* essential oil brand used in this study.

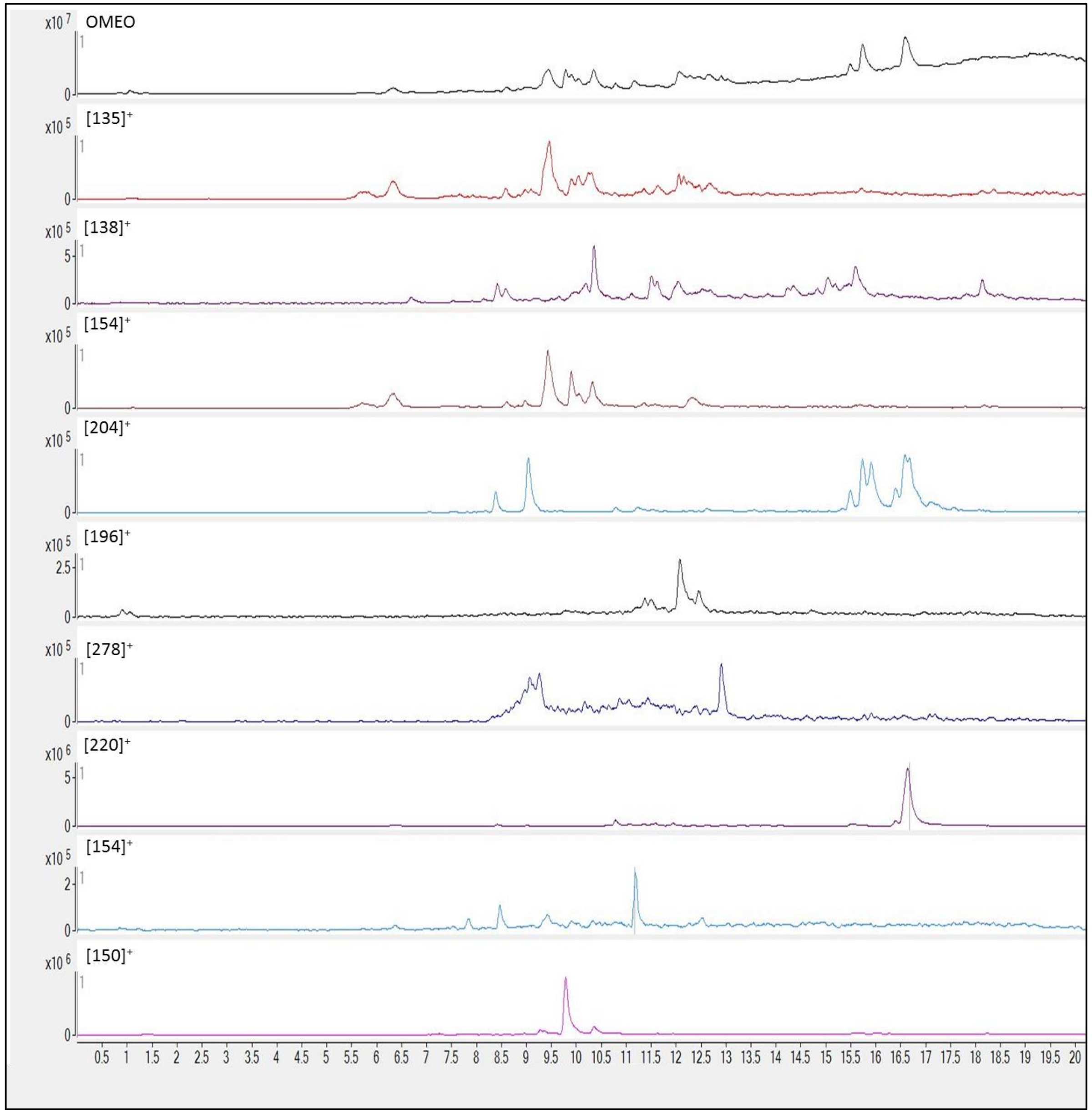

Mass	Possible Compounds
135	Alpha-Pinene, Camphene, p-Cymol, alpha-terpinene
138	Cis-sabinene hydrate
150	Pinocarvone
154	Terpinen-4-ol, alpha-Terpinol
155	P-Meth-2-en-l-ol, Borneol
196	Terpenyl-acetate, Linalyl-acetate
204	B-Caryophyllene, Bicyclogermacrene, Viridoflorene
220	Spathulenol, Caryphyllene oxide
278	Neophytadiene

Supplementary Figure 2. Chromatograms **(A)** and names and mass of compounds **(B)** identified in OMEO by HPLC-MS.
